# Spontaneous relapse in patients with inactive chronic hepatitis B virus infection

**DOI:** 10.22088/cjim.9.4.393

**Published:** 2018

**Authors:** Mojhde Karajibani, Mohammad Reza Hasanjani Roushan, Masomeh Bayani, Mostafa Javanian, Jila Masrour-Roudsari

**Affiliations:** 1 Department of Infectious Diseases, Babol University of Medical Sciences, Babol, Iran; 2 Infectious Diseases and Tropical Medicine Research Center, Babol University of Medical Sciences, Babol, Iran

**Keywords:** Relapse, Hepatitis B, Chronic, Virus, infection

## Abstract

**Background:**

Chronic hepatitis B virus infection (HBV) may reactivate during the course of the disease and is called spontaneous relapse. The purpose of this study was to evaluate the incidence of relapse of hepatitis in subjects with inactive HBV carriers.

**Methods:**

This follow-up study was performed on 785 patients with inactive HBV carriers that were followed-up at six month intervals. The presence of serum HBsAg and anti-HBe, without HBeAg, HBV DNA levels <2000 IU/ml with normal alanine aminotransferase (ALT) levels was defined as inactive carriers. Patients who developed ALT ≥80 IU/L with HBV DNA levels ≥2000 IU/ml were considered as spontaneous relapse.

**Results:**

Seven hundred- eighty five cases (441 males, 344 females) of chronic HBV infected individuals were followed-up. The mean age at the entrance of the study was 30.5±11.8 years. The mean follow-up duration was 5.9±5 years. Relapse was seen in 35 (4.5%) cases, in 27 out of 441 (6.1%) males and in 8 out of 344 (2.3%) females and in 4.2% subjects ≥30 years versus in 4.7% cases of under 30 years (p>0.05). The development of relapse in males was higher than females (hazard ratio 2.53, 95% CI 1.2-5.6, p=0.021), but age ≥30 or <30 years did not have effect (hazard ratio1.21, 95% CI 0.62-2.36, p=0.58).

**Conclusions:**

The results show that spontaneous relapse of hepatitis may develop during the course of chronic HBV infection. We suggest that all patients with chronic hepatitis B, regardless of their age, be examined for the possibility of relapse.

More than 2 billion people in the world were exposed to hepatitis B virus (HBV) infection and 340 million subjects became chronically infected with HBV (1, 2). After chronic HBV infection, the natural course passes into five phases including immune tolerant HBeAg positive phase, immune reactive HBeAg positive phase, inactive carrier state, HBeAg negative reactivation phase, and HBsAg negative phase (3). Among these five phases, patients in immune reactive HBeAg positive phase, and HBeAg negative reactivation phase, should be treated with appropriate anti-viral agents but other phases should be followed periodically for observing the behavior of the virus in these exposed patients.The clinical course of each phase is different from each other and should be followed properly. During inactive carrier state phase, serum HBV DNA levels low (<2000IU/ml), and ALT levels are below the ULN (3). During the course of inactive carrier state, some patients may serocleared of HBsAg associated with the appearance of anti-HBs, and some cases may develop hepatocellular carcinoma and some of them persist this phase indefinitely (4). 

The most important issue during this course is finding spontaneous reactivation of hepatitis (3, 5). Since chronic hepatitis B virus infection (HBV) may reactivate during the course of the disease, the purpose of this study was to evaluate the incidence of relapse of hepatitis in subjects with inactive HBV carriers.

## Methods

From April 2005 to September 2016, 785 patients with chronic HBV who refer to infections clinic of Ayatollah Rouhani Hospital were selected and followed-up at six months. Individuals, who had HCV ab positive or cirrhosis or liver cancer and did not refer for any reason or did not do the necessary tests, were excluded from the study. The presence of serum HBsAg and anti-HBe, without HBeAg, HBV DNA levels <2000 IU/ml with normal alanine aminotransferase (ALT) levels in three occasions 12 months apart was defined as inactive carriers (6, 7). 

These cases were followed-up at six month intervals regarding HBsAg, anti-HBs, ALT, α-fetoprotein and liver sonography. HBV DNA was assessed every two years. Patients who developed ALT ≥80 IU/L with HBV DNA levels ≥2000 IU/ml were considered as spontaneous reactivation of the virus. The viral markers were tested in enzyme-linked immunosorbent assay (ELISA) (HBsAg, from Bio Merieux, France; anti-HBs from Radim Italy, hepatitis B e antigen [HBeAg], two different kits produced from Dia.Pro Diagnostic BioProbes, Italy). 

In these cases, serum HBV DNA levels were measured. For the isolation of HBV DNA, we used the QIAamp DNAkit (Qiagen, Germany). All processes were performed according to the manufacturer’s instructions. For the quantification of HBV DNA, we used Rotor-Gene 3000 (Corbett Research, Australia) using the Artus HBV RG PCR kit (Qiagen, Germany). 

According to the manufacturer’s instructions, the sensitivity of the test was 3·8 IU/ml (1 IU= 7 copies/ml). The study was approved by the Infectious Diseases and Tropical Medicine Research Center of Babol University of Medical Sciences and the research ethics committee approved the study. 

**Statistical analysis: **The data were collected and analyzed using SPSS Version 22. T-test was used to compare mean values. The multiple Cox proportional hazards regression model was used to estimate chronic HBV carrier to relapse with the covariates, sex, and age <30 or ≥30 years. The time to persistence of chronic HBV infection without development of relapse data was plotted using a Kaplan–Meier graph. The log rank test was used to compare the persistence of HBV carriers to development of relases with the same covariates. Differences with a p-value of <0.05 were considered significant. All P-values are two-tailed.

## Results

During the study period, 785 cases (441 males, 344 females) of chronic HBV infected individuals were selected and followed-up. 

The mean age of the patients at the entrance of the study was 30.5±11.8 years.The mean years of follow-up were 5.9±5 years. During the follow-up period, relapse was seen in 35 (4.5%) cases. 

The mean age of the relapse cases was 32.3±10.7 years. Relapse was seen in 8 out of 344 (2.3%) females and in 27 out of 441 (6.1%) was males. Among the 785 cases at the entrance of the study, 358 (45.6%) cases were over 30 years of age. Relapse was seen in 4.2% subjects ≥ 30 years versus in 4.7% cases of under 30 years (p>0.05) (table 1). 

**Table1:** Comparison of healthy carriers Anti Hbe+ by age and gender

**variable**	**Relapse** **N (%)**	**No relapse** **N (%)**	HR:95%CI, p value
**Sex **			
Female Male	8 (2.3%) 27(6.1%)	336(97.8%) 414(93.9%)	HR*: 2.53, 95%CI 1.2-5.6, p=0.021
**Age(year) **			
≥ 30 < 30	15(4.2%) 20(4.7%)	343(95.8%) 407(95.3%)	HR** 1.21, 95%CI 0.62-2.36, p=0.58

* Male/Female

** age<30/≥30 The Data were Analyzed with t-test

**Figure 1:**
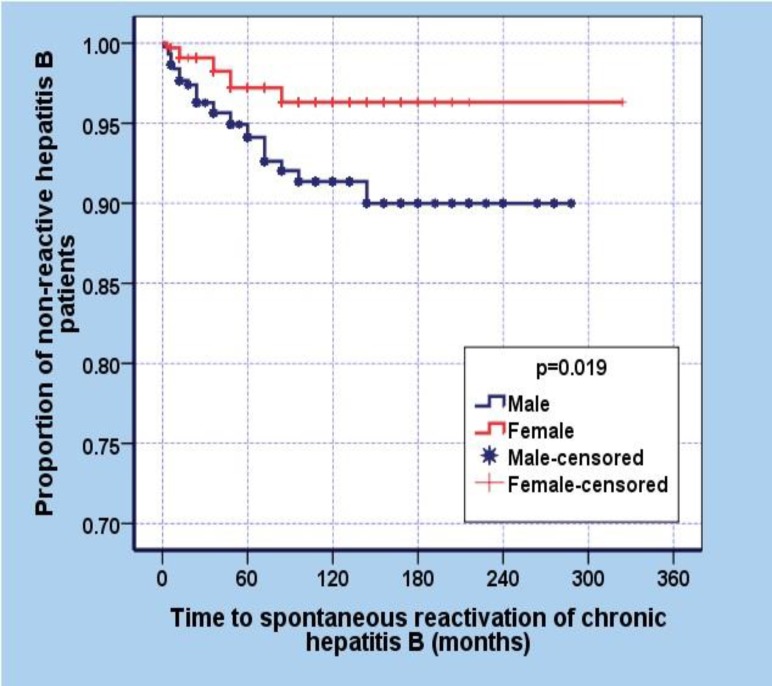
Estimated time of Anti HBe+ healthy carriers relaps by gender

Survival analysis showed that the mean time for the lack of the development of relapse was 303.9 months (95% CI, 296.8-310.9). The mean time in the males was 264.9 (95%CI, 256-273) and for females was 313 (95% CI, 306-321) months (p=0.019) (figure 1). 

Cox regression model showed the development of relapse in males was 2.53 times higher than females (HR=2.53, 95%CI, 1.2-5.6, P=0.021), but the age ≥30 or < 30 years had no effect (HR=1.21, 95% CI, 0.62-2.36, P=0.58). 

## Discussion

In this study, the relapse rate in anti HBe + carriers was 4.5% which was similar to the Kumar study. Follow-up of asymptomatic anti-HBe positive patients to find out those who has relapse and anti-viral therapy is necessary. Kumar et al. in India followed-up 217 cases of asymptomatic anti-HBe positive patients for a median follow-up of 69 months and found that ALT flared up in 43 cases (annual rate of 4.3%). They showed that age >30 years at presentation, male sex, and presence of precore mutants were correlated with ALT flare-up (8). It is estimated that about 300 million individuals are in this phase. More recently, the term “inactive carrier state” has been proposed due to the possibility of reversing this condition. Long-term follow-up studies showed that the vast majority has a sustained biochemical remission and very low risk of progression to cirrhosis or hepatocellular carcinoma (HCC) (9, 10). Hardly, some patients can develop HCC and up to 34% of these patients may have a spontaneous reactivation during follow-up, with or without sero-reversion to HBeAg. The risk of reactivation is higher in the following years after HBeAg seroconversion and decreases with time, although it can be present after years of inactivity. The occurrence of multiple episodes of reactivation or sustained reactivation can cause progressive liver damage and even hepatic decompensation (11, 12). In Portugal, Magalhaes and Pedroto followed-up 100 cases of inactive carriers for 4.6 years and found reactivation in 10% of their cases (13). Some studies reported that HBV reactivation occurred in 10-34% of their patients (14, 15). De Franchis et al followed-up 68 cases of inactive carriers with a mean 10.8 years and found HBV reactivation in 4.4% of their cases (7). 

Villeneuve et al. followed 200 cases in the Canada and follwoed them up in 16 years and found HBV reactivation in the 0.5% of their cases (16). Manno et al. in Italy followed-up 296 cases with a mean follow-up of 30 years and found 2.1% HBV reactivation (17). It is believed that immunological control of the infection by these cases is associated with good long term prognosis with low risk of developing cirrhosis and hepatocellular carcinoma (16, 18, 19). In our study, we have not found any case of cirrhosis or hepatocellular carcinoma. Other researchers also reported a few cases of HCC in their cases and they believed that was related to cofactors like alcohol drinking and metabolic disorders (18). 

In our study, no cases used alcohol consumption due to our advisement for not drinking of this food due to harmful for them. In conclusion, the results show that the spontaneous relapse of hepatitis may develop during the course of chronic HBV infection. Relapse in males was higher. Age ≥30 or <30 years had no effect for the development of relapse.

Not referring the patients due to lack of knowledge of the dangers of the disease, the lack of access to patient’s contact numbers and the cost of the tests were the limitations of the study. Due to the financial limitations, the measurement of viral load was not done in some patients. 

In conclusion, although the rate of relapse in anti HBe^+^ healthy carriers is low, however, considering the risk of relapse and irreparable complications, it is important to follow-up the healthy carriers, specially in the first years of the disease. 
